# Bioactive Compounds Enhance the Biocompatibility and the Physical Properties of a Glass Ionomer Cement

**DOI:** 10.3390/jfb15110332

**Published:** 2024-11-07

**Authors:** Aline Rogéria Freire de Castilho, Pedro Luiz Rosalen, Marina Yasbeck Oliveira, Jonny Burga-Sánchez, Simone Duarte, Ramiro Mendonça Murata, Regina Maria Puppin Rontani

**Affiliations:** 1 Department of Pediatric Dentistry, Indiana University School of Dentistry, 1121 W. Michigan St., Indianapolis, IN 46202, USA; 2 Departamento de Biociências, Faculdade de Odontologia de Piracicaba, Universidade Estadual de Campinas, Piracicaba 13414-903, SP, Brazil; rosalen@fop.unicamp.br (P.L.R.); marina.yasbeck@gmail.com (M.Y.O.); jonnyburga@gmail.com (J.B.-S.); 3 Faculdade de Ciências Farmacêuticas, Universidade Federal de Alfenas, Alfenas 37130-001, MG, Brazil; 4 School of Dental Medicine, University at Buffalo, Buffalo, NY 14215, USA; simonedu@buffalo.edu; 5 Department of Foundational Sciences, School of Dental Medicine, East Carolina University (ECU), Greenville, NC 27834, USA; muratar16@ecu.edu; 6 Departamento de Ciências da Saúde e Odontologia Infantil, Faculdade de Odontologia de Piracicaba, Universidade Estadual de Campinas, Piracicaba 13414-903, SP, Brazil; rmpuppin@unicamp.br; 7 Departamento de Odontologia Restauradora, Faculdade de Odontologia de Piracicaba, Universidade Estadual de Campinas, Piracicaba 13414-903, SP, Brazil

**Keywords:** flavonoids, dental materials, glass ionomer cements, surface properties, toxicity

## Abstract

In order to characterize a novel restorative material, knowledge about the toxicological effect on human cells and the physical behavior of a glass ionomer cement (GIC) containing flavonoids was established. The flavonoids apigenin, naringenin, quercetin, and liquiritigenin were manually incorporated into a GIC. In the control group, no incorporation was performed. Two cell culture assays evaluated the toxicity of GICs: SRB and MTT. For both assays, the keratinocyte cell line (HaCaT) was exposed to GIC (*n* = 3/group) for 24 h. The physical properties of the GICs were evaluated by compressive strength (*n* = 10), surface roughness (*n* = 10), and hardness (*n* = 10) tests. Cell viability by SRB ranged from 103% to 97%. The control revealed a significant decrease in the metabolism of cells (61%) by MTT, while the GIC+apigenin slightly increased the succinic dehydrogenase activity (105%; *p* > 0.05), also confirmed microscopically. The compressive strength and roughness values were similar among groups, but the hardness increased after the incorporation of naringenin and quercetin into GIC (*p* < 0.05). The incorporation of flavonoids positively altered the biological and physical properties of the GICs.

## 1. Introduction

Conventional glass ionomer cement (GIC) stands as a cornerstone in restorative dentistry, particularly in pediatric patients with high caries risk, due to its fluoride release and chemical bonding to dental surfaces in moist environments [1,2]. However, the clinical utility of conventional GIC is constrained by its inherent low mechanical strength, limiting its application to non-stress-bearing areas [3].

To address this limitation, strategies to reinforce GIC were investigated, with a particular focus on incorporating bioactive compounds, such as nanoparticles, antimicrobials, and plant-based natural products [4,5,6,7,8]. These modifications aim not only to improve the physical properties of GIC but also to introduce bioactive capabilities that can contribute to long-term clinical success [9]. Bioactive compounds enhance the GIC by promoting remineralization, antibacterial effect, and overall biological compatibility, making it an ideal candidate for high-risk patients [10]. Flavonoids, a diverse group of plant secondary metabolites, possess an array of pharmacological properties, including antimicrobial, anti-inflammatory, and antioxidant activities [11]. Among these, apigenin, quercetin, naringenin, and liquiritigenin have garnered attention for their potential to enhance the performance of dental materials. These natural compounds, when incorporated into GIC, could not only improve its physical properties but also introduce bioactivity that could significantly enhance the therapeutic outcomes of restorative treatments [10,12].

Apigenin, for instance, demonstrates inhibitory effects on glucosyltransferases (GTFs), crucial enzymes involved in biofilm formation by *Streptococcus mutans*, thus modulating virulence factors associated with dental caries [13]. Similarly, quercetin’s antioxidant properties promote biomineralization on dentin surfaces while also fostering bone tissue regeneration and mitigating oral inflammation [14,15]. Naringenin, with its antioxidant and anti-inflammatory attributes, exhibits promising effects on inhibiting biofilm formation, further underscoring its potential in dental applications [16]. Additionally, liquiritigenin offers strong antimicrobial and anti-inflammatory effects, making it a viable candidate for enhancing the therapeutic properties of dental materials [17].

Incorporating flavonoids into GIC represents a promising strategy for improving its physical properties, such as compressive strength, roughness, and hardness, while simultaneously leveraging the therapeutic benefits of these bioactive compounds. Additionally, the bioactive properties of flavonoids can enhance the antibacterial and anti-inflammatory capabilities of GIC, contributing to the material’s ability to control secondary caries, support tissue healing, and increase overall longevity. Therefore, it is imperative to ensure that such modifications do not cause adverse interactions with oral tissues, without a significant level of cytotoxicity of the material, maintaining its suitability for clinical use. Thus, this paper aimed to provide a comprehensive understanding of the physical properties of flavonoid-containing GICs and their toxicological effects on human cells, shedding light on their potential as advanced dental restorative materials.

## 2. Materials and Methods

For this study, a conventional glass ionomer cement (GIC; GC Gold Label 9, Batch #1212051) was modified by manually adding four different flavonoids: apigenin (39.67 µM; 0.0106%); liquiritigenin (0.23 µM; 0.00065%); naringenin (0.48 µM; 0.00131%); and quercetin (20.15 µM; 0.0609%) (Sigma–Aldrich, Steinheim, Germany) [6,7,8]. Concentrations used in the current study were previously determined by in vitro antimicrobial assays [18]. GIC with no flavonoid was used as the control.

### 2.1. Specimen Preparation

Cylindrical specimens (5 mm × 2 mm) containing or not containing flavonoids were prepared, according to the manufacturer’s instructions, by using silicone molds at a temperature of 23 °C ± 1 °C and a relative humidity of 50% ± 5%. Specimens were superficially protected with petroleum jelly and stored at 37 °C and 100% relative humidity for 24 h for surface assays. Afterward, each specimen surface was polished using Sof-Lex discs (3M ESPE, St. Paul, MN, USA), washed through sonication for 10 min, soaked in 70% ethanol for 1 min for disinfection, dried, and exposed to UV radiation for physical sterilization before cytotoxicity and physical tests [6,7,8,18].

### 2.2. Cell Culture Process

Prior to use, the spontaneously immortalized human keratinocyte (HaCaT- Human adult low Calcium high Temperature keratinocytes) cells (Bank of Cells of Rio de Janeiro, Rio de Janeiro, Brazil) were maintained in a monolayer Dulbecco’s Modified Eagle’s Medium (DMEM; Sigma Chemical Co., St. Louis, MO, USA) supplemented with 10% fetal bovine serum (FBS; Gibco, Grand Island, NY, USA), 100 IU/mL penicillin, and 100 mg/mL streptomycin (Vitrocell Embriolife, Campinas, SP, Brazil) in a humidified incubator with 5% CO_2_ and 95% air at 37 °C (Isotemp; Fisher Scientific, Pittsburgh, PA, USA). Cells were subcultured every 3 days until an adequate number of cells was reached. Then, the cells were seeded (5 × 10^4^ cells/well) in sterile 24-well plates (Costar Corp., Cambridge, MA, USA) and incubated at 37 °C for 48 h with 5% CO_2_ and 95% air [6,18].

#### 2.2.1. Cell Culture Assays

##### Cell Viability by Protein Basic Residues Density (Sulforhodamine B Assay-SRB)

Cellular protein content was assessed by colorimetric SRB assay. Briefly, specimens of experimental and control groups (*n* = 3) were inserted separately into wells containing DMEM medium for 24 h. Then, the cells were washed with phosphate-buffered saline solution (PBS; Gibco, Grand Island, NJ, USA; pH 7.4), and the cellular proteins were fixed by adding 10% trichloroacetic acid (TCA; Sigma–Aldrich, Steinheim, Germany) and incubated for 60 min at 4 °C. The supernatant was discarded, and plates were rinsed with deionized water and then stained with 0.4% SRB dye solution (Sigma–Aldrich, Steinheim, Germany) in 1% glacial acetic acid for 15 min at room temperature. Unbound SRB was removed by washing with 1% acetic acid before air drying. The bound stain was solubilized with 10 mM Tris buffer (UltraPureTM Tris, Invitrogen, Waltham, MA, USA; pH 10.5), and the optical densities were read on an automated spectrophotometric plate reader (ASYS UVM340, Biochrom Ltd., Cambridge, UK) at a single wavelength of 540 nm. Three independent assays were performed for the analysis [18,19].

##### Cell Proliferation and Function Assay at the Mitochondrial Level (Vybrant^®^ MTT Proliferation Assay)

In order to evaluate metabolic cell function and proliferation capability, we used a colorimetric MTT Cell Proliferation Assay kit (Molecular Probes Life Technologies, Carlsbad, CA, USA). Briefly, 5 × 10^4^ cells/well were seeded in a 24-well plate with DMEM medium supplemented with 10% FBS and exposed to specimens. To quantify cell metabolic activity, after 24 h, DMEM medium containing MTT 0.3 mg/mL was added in each well, and cells were incubated for 3 h in 95% air and 5% CO_2_ at 37 °C. Absorbance was measured at 570 nm in a microspectrophotometer (ASYS UVM340, Biochrom Ltd., Cambridge, UK). Three independent assays were performed for the analysis [6,18].

#### 2.2.2. Analysis of HaCaT Cell Morphology by Microscopy

HaCaT cell morphology images were taken immediately before exposure to specimens and 24 h after incubation using an optical microscope (Zeiss Axiovert 40 CFL, Oberkochen, BW, Germany) at a magnification of 10×.

#### 2.2.3. Live/Dead^®^ Viability/Cytotoxicity Assay

To simultaneously analyze cell function and viability, a Calcein/AM-Ethidium homodimer-1 Viability/Cytotoxicity assay kit (Live/Dead^®^, Molecular Probes Life Technologies, Carlsbad, CA, USA) was also used. For this purpose, 1 × 10^4^ cells/well were seeded in a 24-well plate, incubated for 24 h, and exposed to the specimens (*n* = 3/group) for 24 h. The Live/Dead^®^ assay was performed following the manufacturer’s recommendations. After exposure to the specimens, the cells were washed twice with PBS, followed by the addition of 300 μL of the Live/Dead^®^ solution to each well containing HaCaT cells. Fluorescence images were analyzed using an inverted microscope (Axiovert 40 CFL, Carl Zeiss, Germany) coupled to an MEC camera (AxioCam, Carl Zeiss, Germany). Calcein/AM was detected using a wavelength range of 450–490 nm (excitation) to 515–565 nm (emission). EthD-1-labeled cells (dead) were detected using wavelengths of 528–546 nm (excitation) and 590–617 nm (emission) [6,18].

### 2.3. Physical Properties

Specimens (*n* = 10) were prepared for each physical assay, including compressive strength, surface roughness, and surface microhardness, following the preparation methods outlined above [6]. The tests were conducted in accordance with ISO 9917–1:2007 [20] for water-based cements. This ISO standard ensures that the testing methods are consistent with internationally recognized protocols for evaluating the physical properties of dental materials.

The sample size (*n* = 10) was selected to ensure statistical significance, based on previous studies and literature. Using a minimum of 10 specimens per group allowed for reliable results, reducing variability and enabling meaningful comparisons between the experimental GICs and the control group. All specimens were prepared with identical dimensions, as described for each assay, to maintain uniformity and reduce experimental bias [6,7,8,18].

#### 2.3.1. Compressive Strength

Compressive strength was measured using an Instron universal testing machine (Model 4411, Instron Co., Canton, MA, USA). Specimens were cylindrical (5 mm height × 2 mm diameter) to meet the sample geometry standards provided by the ISO, ensuring consistent load application and measurement accuracy. A crosshead speed of 1.0 mm/min was applied until fracture. This test simulated clinical force on the GIC restorations and helped evaluate the material’s mechanical performance under stress [6,8,18].

#### 2.3.2. Roughness Measurements

Specimens (5 mm height × 2 mm diameter) were fitted to a roughness-measuring instrument (Surfcorder SE1700, Kosaka Corp, Tokyo, Japan), following ISO 4287 [21] guidelines, which specifies methods for roughness measurements in dental materials. To record the roughness measurements, the instrument’s needle moved at a constant speed of 0.5 mm/sec with a load of 0.7 mN. The cut-off value was set at 0.25 mm. The roughness profile (Ra) was recorded across the diameter of each specimen, with three readings taken per specimen to ensure accuracy. This test was critical for assessing the surface quality of the GICs, as roughness influences the material’s resistance to plaque accumulation and wear [6,8,18].

#### 2.3.3. Hardness Measurements

Surface microhardness was assessed using a Shimadzu HMV-2000 microhardness tester (Shimadzu Corporation, Kyoto, Japan) in accordance with ISO 4545 [22] standards for the Knoop hardness test. A load of 25 g was applied for 15 s to the surface of each specimen (5 mm height × 2 mm diameter), and three indentations were made at 100 μm intervals. The mean microhardness value was calculated for each specimen [6]. This setup allowed for the precise measurement of surface hardness, a critical factor in evaluating the resistance of GIC to surface deformation and wear over time [6,7,8,18].

### 2.4. Statistical Analysis

The normality of the cell viability data was assessed using the Shapiro–Wilk test, which evaluates whether the data follows a normal distribution. This test is particularly effective for small sample sizes and is commonly used in biological data analysis. Homogeneity of variances was confirmed using Levene’s test, which ensures that the variances across the groups being compared are equal, a necessary condition for parametric tests such as ANOVA. Once normality and homogeneity of variance were confirmed, a one-way ANOVA was performed to determine if there were any statistically significant differences among the groups. Following the ANOVA, a Tukey post hoc test was applied to pinpoint which specific groups differed from each other.

For the physical properties data, which included compressive strength, surface roughness, and microhardness, a one-way ANOVA was also used to detect any significant differences among the experimental and control groups. Similarly, the Tukey post hoc test was employed to identify where the specific differences lay between the groups. All statistical analyses were carried out using the GraphPad Prism^®^ 6.0 software package. The significance level for all tests was set at *p* < 0.05, indicating that differences with a probability of less than 5% were considered statistically significant.

## 3. Results

### 3.1. Cell Culture Assays

#### 3.1.1. SRB Assay

**Figure 1:**
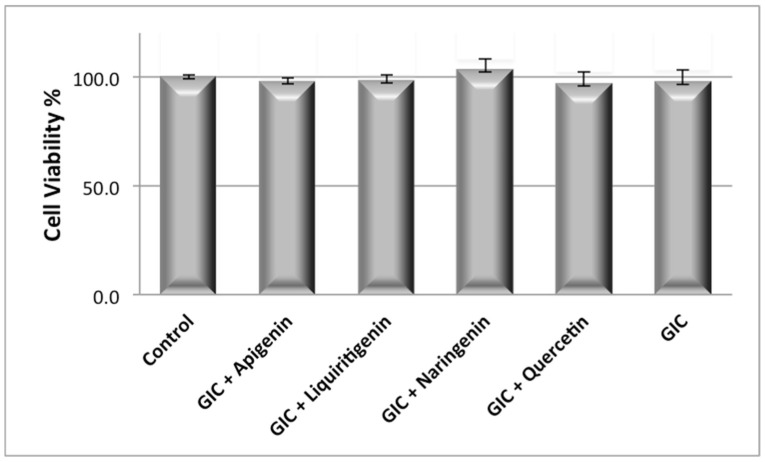
Effect of GIC containing or not containing flavonoids on HaCaT cell viability after 24 h exposure by SRB assay. All results are normalized to a maximum value of 100% using a positive control. Data shown in the figure are mean ± SD, with *p* > 0.05 from assays performed in triplicate.

Considering the negative control as 100% of the cell viability, the percentage of cell viability observed during the SRB assay for experimental groups ranged from 103.1% (GIC + naringenin) to 97% (GIC + quercetin). After 24 h of treatments, the GIC containing no flavonoids induced toxic effects to the HaCat cells and were not significantly different from the negative control (*p* > 0.05) (Figure 1).

#### 3.1.2. MTT Assay

**Figure 2:**
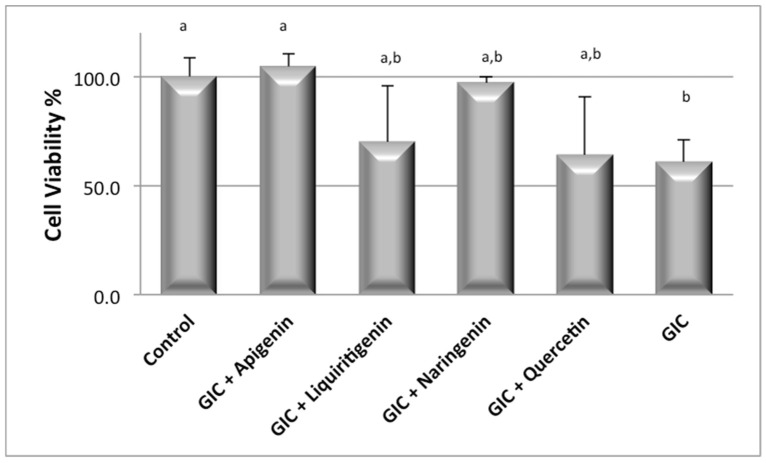
Metabolic activity determination on HaCaT exposed to GIC containing or not containing flavonoids. Mean MTT quantification results obtained for 24 h incubation time. All results are normalized to a maximum value of 100% using a positive control. Data shown in the figure are mean ± SD, from assays performed in triplicate. Statistically significant differences are labeled with different lower-case letters (e.g., a/b).

Figure 2 shows the cell function and proliferation following exposure to GIC with or without flavonoids during the MTT assay. The GIC (positive control) caused a significant decrease in the metabolism of HaCat cells (61%) when compared to the negative control (100%) and GIC + apigenin (*p* < 0.05). The GIC + apigenin slightly increased the succinic dehydrogenase (SDH) activity (105%), compared to the control (*p* > 0.05). Liquiritigenin and quercetin slightly decreased cell proliferation, but their biocompatibility did not differ from control groups. There was no significant difference amongst the remaining groups, indicating that GICs containing flavonoids have a low toxicity profile.

#### 3.1.3. Microscopic Analysis and Live/Dead^®^ Viability/Cytotoxicity Assay

**Figure 3:**
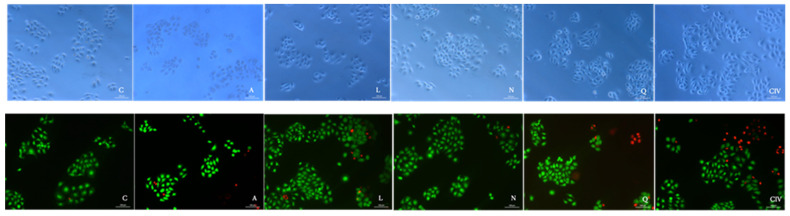
Illustrative images of HaCaT cells exposed for 24 h to GIC containing or not containing flavonoids analyzed by using optical microscopy (first line) and Live/Dead^®^ assay (second line), represented by C—control (HaCat cells); A—GIC + apigenin; L—GIC + liquiritigenin; N—GIC + naringenin; Q—GIC + quercetin; and GIC—glass ionomer cement (without flavonoids). Green cells correspond to live cells, whereas dead cells are stained in red. This was performed at 10× magnification, scale bar = 100 μm.

Morphological characteristics of normal keratinocytes, such as size and polyhedral shape, arranged in contact between them were identified. As the enzymatic activity of cytoplasmic esterases, originating from living cells, on calcein produces an intense green color, this color labeled cells that showed viability. This color was intensely observed in all groups (Figure 3).

Changes in cell shape, from polyhedral to rounded, were observed in very few HaCaT cells exposed to GICs with or without flavonoids. The bright red color, a product of ethidium homodimer-1 binding to nucleic acids, and loss of cell contact, which is indicative of cell death, were observed after 24 h of treatment.

### 3.2. Physical Properties

The means and standard deviations of the compressive strength, roughness, and hardness are shown in Table 1. No significant differences were observed amongst groups for the compressive strength and roughness, but the hardness surface was increased after the incorporation of naringenin and quercetin (*p* < 0.05), showing that the incorporation of flavonoids may positively modify the original properties of the GIC.

## 4. Discussion

In recent years, significant attention has been focused on exploring plant-based natural products as potential bioactive therapies for dental caries, drawing upon traditional medicinal knowledge [23]. However, despite these endeavors, the safety and physical properties of isolated compounds associated with GIC remain largely unexplored. The selection of flavonoids was guided by their documented pharmacological properties, with the concentration chosen based on previous research highlighting their antimicrobial efficacy.

Given that it is imperative to evaluate the potential toxicity of emerging dental products before clinical application, in vitro cytotoxicity assays serve as valuable tools for predicting safety and potential toxicity during the early stages of research [24]. The MTT assay remains one of the most useful and popular tests for evaluating cell viability following exposure to toxic substances [25]. Essentially, MTT involves the conversion of the water-soluble MTT (3-(4,5-dimethylthiazol-2-yl)-2,5-diphenyltetrazolium bromide) to an insoluble formazan in the mitochondria [25]. In contrast, the SRB cell cytotoxicity assay is an easily reproducible colorimetric assay based on the ability of SRB to bind to protein basic amino acid residues, providing a sensitive indicator of cellular protein content that is linear over a cell density range [25]. Currently, the SRB assay is the key assay adopted by the National Cancer Institute (NCI) in the USA [26]. It exhibits high sensitivity, independent of cell metabolic activity, along with flexible time measurements [27]. Essentially, MTT and SRB assays employ different physiological mechanisms, which may yield differing results [28].

Overall, the results from cell culture assays (SRB assay, MTT assay, and microscopic analysis) indicate that GICs containing flavonoids, such as apigenin, liquiritigenin, and quercetin, demonstrate enhanced biocompatibility, compared to traditional GIC formulations. This observation is crucial for dental applications, where materials in direct contact with oral tissues must exhibit minimal cytotoxicity and support cell viability. In general, findings highlighted that in the MTT assay, cell function and proliferation of liquiritigenin, naringenin, and quercetin associated with GIC were lower, indicating a low toxicity profile for these groups, while apigenin slightly stimulated cell proliferation. However, it was not significantly different from the negative control, suggesting that GIC associated with flavonoids presented no toxicity. SRB, in contrast, provided a sensitive index of cellular protein content that was linear over a cell density range after 24 h of exposure to GIC specimens. The stability of the SRB assay’s outcome was ensured by fixing the drug-exposed cells with TCA after incubation [16,27]. Following cell fixation and staining, minimal changes in SRB optical densities (less than 2%) may be observed over a 7-day period if evaporation is prevented. Moreover, the SRB assay provided superior signal-to-noise values, even with low cell concentrations [29]. Additionally, several limitations of the MTT assay were addressed [28]. A previous report highlighted the highest variability of MTT, suggesting inaccuracies in detecting changes in the cell number, as well as a high variation in glycolysis inhibitor concentrations [28]. Furthermore, staining in the MTT assay was cell-line dependent [30] and less sensitive when cell numbers were below 1000 cells/well [28]. Therefore, careful consideration of data was necessary to avoid misinterpretation. Therefore, based on the explanations provided and the current data, the findings of the SRB assay may be considered superior to those of the MTT assay, since the SRB evaluation demonstrated lower variability and thus greater accuracy and sensitivity in detecting changes in cell number, compared to MTT [28].

Despite the incorporation of flavonoids, the GIC formulations did not induce significant toxic effects on keratinocyte cells, as evidenced by cell viability assays. This suggests that the addition of flavonoids does not compromise the safety of GICs and may even improve their biocompatibility profile. The HaCaT cell line includes adherent growing, spindle-shaped, and fibroblast-like flat cells that are filled by the nucleus, creating the appearance of almost a regular epidermal design [31]. The HaCaT cells may express high differentiation capacity and great genetic stability [32]. Images of these cells exposed to GIC containing or not containing flavonoids indicate that the addition of those compounds to the restorative material did not affect the cell viability. After treatment with GICs for 24 h, a high proportion of HaCaT was regularly stained green. The fluorescence assay indicated that the GIC containing apigenin or naringenin exhibited a brighter green color than the other groups, showing a possible protective effect of those flavonoids on cells. The results are in accordance with the MTT (105%) and SRB (103.1%) assays on those groups, respectively.

The analysis of physical properties revealed significant insights into the impact of flavonoid incorporation on the mechanical characteristics of GICs. The increased surface hardness associated with the addition of naringenin and quercetin underscores the potential of flavonoids to positively influence the inherent properties of GICs. This finding is particularly relevant for dental restorative materials, as enhanced physical properties contribute to the durability and longevity of dental restorations. Over the past few decades, various substances have been incorporated into GICs to improve their anticariogenic activity [4,5,6,7,8]. Among these, propolis, a natural resin produced by bees, has emerged as one of the most extensively studied extracts due to its significant pharmacological properties [33]. Some studies have indicated that the addition of propolis to GICs does not affect certain physical properties, such as shear bond strength [34] and microleakage [35]. However, incorporating plant extracts may influence other important characteristics, including microhardness [35], compressive strength [36], fluoride release [37], water sorption, and diametral tensile strength [34], depending on their chemical origin [38]. Green propolis is rich in flavonoids like apigenin, liquiritigenin, naringenin, and quercetin, which contribute to its bioactivity [39]. During the setting process of GIC, the acidic components react with the basic glass powder to form a gel-like matrix [40]. In this context, the bioactive compounds likely interact with the carboxyl groups of the GIC [38], potentially modifying gelation kinetics and altering the physical structure of the matrix [40]. Additionally, the hydrophilic nature of flavonoids may promote better dispersion throughout the cement matrix, ensuring uniform distribution and retention of these additives, thereby enhancing the material’s hardness. This improved interaction could also influence the release profile of the flavonoids, facilitating sustained bioactivity over time. A more comprehensive understanding of these interactions could be explored in future studies to optimize the clinical performance of GIC. The controlled and sustained release of bioactive compounds can prolong the material’s bioactive properties, while a gradual release mechanism ensures that the beneficial effects of the flavonoids are maintained over an extended period, improving the durability and performance of the restoration.

Changes in the compressive strength of GIC after incorporating flavonoids were not observed in this study. Similarly, roughness, a surface property, was also unaffected. These outcomes may be attributed to the use of isolated flavonoid compounds, which might not fully replicate the effects of whole plant extracts. Plant extracts contain a broad spectrum of bioactive compounds, including various flavonoids, phenolics, and other secondary metabolites. The synergistic and antagonistic interactions among these compounds could significantly influence the physical properties of GIC. Consequently, the complex interplay of multiple compounds in plant extracts may alter GIC properties differently, compared to isolated flavonoids, which do not capture the full range of potential interactions. Surprisingly, the incorporation of the flavonoids naringenin and quercetin improved the hardness of the material. This increase in hardness may be attributed to the interaction of flavonoids with the GIC matrix, potentially influencing the cross-linking density and overall structural integrity. Since modifications to GIC should not negatively impact its inherent properties, we infer that the amount of bioactive agent added did not compromise the powder/liquid (P/L) ratio [41]. Maintaining the P/L ratio ensures that the acid–base reaction between polyacrylic acid and fluoroaluminosilicate glass remains intact, which is critical for the setting reaction and the physical strength of the GIC [40]. Given that flavonoids enhance surface hardness, their effects on wear resistance and setting time are likely minimal. Understanding these effects is crucial for optimizing GIC formulations to address the varied clinical requirements of dental practitioners.

Interestingly, Hu et al. [42] examined the impact of incorporating a flavonoid, epigallocatechin-3-gallate (EGCG), into GIC and observed an enhancement in the quality of this restorative material [42]. The addition of EGCG to GIC resulted in increased flexural strength and surface microhardness, though it did not affect fluoride release [41]. This finding is consistent with our results, as observed with naringenin and quercetin, which also increased surface hardness. Several factors may contribute to this increase: (1) attention to specimen preparation is essential to maintaining proper consistency and setting time; (2) the molecular weight of the compound may also influence the molecular weight of polyacrylic acid, affecting the rate of the acid–base reaction of GICs and thereby modifying physical properties [41]; and (3) the concentration of flavonoid molecules on the GIC surface [41]. While not the focus of this study, we identified a limitation in the use of quercetin, as its incorporation caused the color of GIC to turn brownish; thus, this material may be less clinically acceptable. However, it could be an intriguing alternative for restorative treatment decisions on posterior teeth, where close supervision of fillings is necessary to avoid secondary caries lesions.

The integration of plant-based natural products into dental materials presents a promising avenue for enhancing their therapeutic properties. While much attention has been directed towards exploring these substances for their potential in combating dental caries, there remains a significant gap in our understanding of their safety and impact on the physical properties of restorative materials, such as GIC. Our study sheds light on the potential benefits of incorporating flavonoids into GIC, highlighting their effects on cell viability and material properties. The enhanced biocompatibility and hardness conferred by flavonoid additives suggest the potential for developing novel GIC formulations with improved clinical performance and patient outcomes. However, further research is needed to fully elucidate the implications of these findings and to optimize the use of bioactive compounds in dental applications.

While our study provided valuable insights into the effects of flavonoids on the biocompatibility and physical properties of glass ionomer cement (GIC), several limitations should be acknowledged. First, our study focused primarily on the in vitro effects of flavonoid incorporation, particularly on cell viability and GIC’s physical properties. Although these results are promising, in vivo studies are essential to assess the long-term performance, biological interactions, and durability of these modified GICs under the complex conditions of the oral environment. Additionally, our study utilized a limited number of flavonoids, and further research is needed to explore other plant-based bioactive compounds, which may provide additional therapeutic benefits or have synergistic effects when combined with current additives.

Another limitation of the current study is the relatively narrow scope of the physical tests performed. While compressive strength, surface roughness, and hardness were evaluated, more comprehensive testing of other key properties, such as wear resistance, fatigue strength, and fluoride release, is necessary to fully assess the potential of flavonoid-containing GICs for clinical use. Moreover, the impacts of these bioactive compounds on the material’s setting reaction, bonding strength, and long-term stability should be evaluated to ensure the practicality of these modified materials in stress-bearing areas. Further work is also needed to address the potential aesthetic limitations associated with flavonoid incorporation, as the observed color changes, particularly with quercetin, may affect the clinical acceptability of these materials in aesthetic zones. Overall, continued investigation into the integration of natural products into dental materials holds promise for advancing dental treatment and improving patient outcomes.

## 5. Conclusions

Taken together, the outcomes of this study demonstrate that the biocompatibility of GIC to keratinocyte cells was improved by the presence of flavonoids. Additionally, flavonoid-containing GIC might be an effective strategy in dentistry, as their surface hardness was enhanced by flavonoids without modifying other physical properties. Further studies will provide information about cell viability and proliferation in a dose-dependent relationship after the exposure of cells to these materials, as well as their influence on other physical properties.

## Figures and Tables

**Table 1:** Roughness, surface hardness, and compressive strength of GIC containing or not containing flavonoids.

Groups		Mechanical Properties	
	Roughness (µM)	Hardness (KHN)	Compressive Strength (MPa)
GIC (no compound)	0.73 ± 0.2 ^a^	44.16 ± 8.0 ^a^	23.87 ± 8.9 ^a^
GIC + Apigenin	0.74 ± 0.4 ^a^	51.60 ± 6.2 ^a^	30.80 ± 10.2 ^a^
GIC + Liquiritigenin	0.61 ± 0.3 ^a^	52.10 ± 7.2 ^a^	21.57 ± 6.9 ^a^
GIC + Naringenin	0.91 ± 0.5 ^a^	60.38 ± 14.6 ^b^	27.42 ± 9.1 ^a^
GIC + Quercetin	0.94 ± 0.4 ^a^	70.85 ± 10.8 ^b^	34.47 ± 13.1 ^a^

For each assay, different lower-case letters (e.g., a/b) indicate mean differences (*p* < 0.05).

## Data Availability

The original contributions presented in the study are included in the article, further inquiries can be directed to the corresponding author.
